# Complete Genome Sequence of *Diaphorina citri-associated C virus*, a Novel Putative RNA Virus of the Asian Citrus Psyllid, *Diaphorina citri*

**DOI:** 10.1128/genomeA.00639-16

**Published:** 2016-07-21

**Authors:** Shahideh Nouri, Nidà Salem, Bryce W. Falk

**Affiliations:** a Department of Plant Pathology, University of California Davis, Davis, California, USA; b Department of Plant Protection, The University of Jordan, Amman, Jordan

## Abstract

We present here the complete nucleotide sequence and genome organization of a novel putative RNA virus identified in field populations of the Asian citrus psyllid, *Diaphorina citri*, through sequencing of the transcriptome followed by reverse transcription-PCR (RT-PCR). We tentatively named this virus *Diaphorina citri-associated C virus* (DcACV). DcACV is an unclassified positive-sense RNA virus.

## GENOME ANNOUNCEMENT

In our previous study, we reported partial sequences of a novel positive-sense, bipartite RNA virus in the Asian citrus psyllid, *Diaphorina citri*, tentatively named *Diaphorina citri-associated C virus* (DcACV) (1). Here, we are reporting the complete nucleotide sequence of DcACV. Two unjoined contigs of 2,279 and 1,645 nucleotides were assembled from the *D. citri* transcriptome and confirmed by reverse transcription-PCR (RT-PCR) followed by Sanger sequencing. The nucleotide sequences of both ends of the genomic RNAs were determined by rapid amplification of cDNA ends (RACE) using the SMARTer 5′/3′ RACE system according to the manufacturer’s instructions (Clontech, Mountain View, CA). The complete sequences of the DcACV genomic RNAs are 2,376 and 1,817 nucleotides (nt) for RNA1 and RNA2, respectively, and both RNAs are nonpolyadenylated. The 5′ and 3′ untranslated regions (UTRs) of RNA1 are 180 and 184 nt, respectively, flanking two predicted overlapping open reading frames (ORFs). ORF1 (nt 181 to 1083) and ORF2 (nt 654 to 2192) encode putative proteins of 300 and 512 amino acids, respectively. RNA2, which contains the 5′ and 3′ UTRs of 127 and 140 nt, respectively, encodes two putative proteins of 161 and 398 amino acids from predicted overlapping ORF1 (nt 128 to 613) and ORF2 (nt 481 to 1677), respectively. The first 17 nucleotides of both 5′ UTRs are identical.

The DcACV ORF2 predicted protein on RNA1 shows significant similarity with viral RNA-dependent RNA polymerases (RdRp) and contains a conserved GDD motif. However, no significant similarities were found between the other DcACV deduced amino acid sequences and the known protein sequences in databases. BLASTp analysis of the putative RdRp encoded by ORF2 of RNA1 showed the highest significant amino acid sequence identity (identity 44%; query coverage 92%) to a recently discovered virus of *Scaptodrosophilla deflexa* named Tartou virus (accession no. AMO03231) (2), which itself is distantly related to viruses of *Tombusviridae*. This was consistent with our phylogenetic analysis based on the RdRp, which placed DcACV close to tombusviruses (1). Conversely, the putative protein encoded by ORF2 of RNA 2 shared the highest significant amino acid sequence identity (identity 39%; query coverage 12%) with the *Chronic bee paralysis virus* (CBPV) (accession no. YP001911140), the causal agent of the chronic bee paralysis disease in honeybees around the world (3). CBPV has not been assigned to any family or genus; however, it occupies an intermediate phylogenetic position between viruses of *Nodaviridae* and *Tombusviridae* (3).

### Nucleotide sequence accession numbers.

The complete genome sequences of DcACV genomic RNAs have been deposited in GenBank under the accession numbers KX235518 and KX235519 for RNA1 and RNA2, respectively.
